# Safety and effectiveness of dabigatran vs rivaroxaban in nonvalvular atrial fibrillation patients at nutritional risk

**DOI:** 10.1016/j.rpth.2026.106865

**Published:** 2026-07-21

**Authors:** Linhao Liu, Hangkuan Liu, Geru Aa, Jingbo Yang, Zhiqiang Zhang, Tianqi Yang, Bin Sun, Lushu Zuo, Haoran Qin, Shuting Liu, Ching-Hui Sia, Pengfei Sun, Qing Yang, Xin Zhou

**Affiliations:** 1 Department of Cardiology, Tianjin Medical University General Hospital, Heping District, Tianjin, China; 2 Department of Cardiology, National University Heart Centre, Singapore

**Keywords:** atrial fibrillation, dabigatran, intracranial hemorrhage, nutritional status, rivaroxaban

## Abstract

**Background:**

Malnutrition is a prevalent comorbidity in nonvalvular atrial fibrillation (NVAF), yet comparative evidence on oral anticoagulants in this vulnerable population is scarce.

**Objectives:**

We aimed to compare the safety and effectiveness of dabigatran with those of rivaroxaban in NVAF patients at nutritional risk.

**Methods:**

This new-user, active-comparator cohort study utilized data from the Tianjin Health and Medical Data Platform (2015-2020). We identified 10,658 NVAF patients (dabigatran *n* = 1819; rivaroxaban *n* = 8839) at nutritional risk, defined as a Prognostic Nutritional Index score ≤50. Multivariable Cox proportional hazards models were used to compare the risks of intracranial hemorrhage (ICH), gastrointestinal bleeding, ischemic stroke, and all-cause mortality over a maximum 3-year follow-up. Extensive sensitivity analyses were performed to ensure the robustness of the findings.

**Results:**

The incidence of ICH was significantly lower in the dabigatran group compared with the rivaroxaban group (0.55% vs 1.27%; hazard ratio [HR], 0.42; 95% CI, 0.22-0.80). This lower risk was particularly evident in patients receiving low-dose regimens (HR, 0.33; 95% CI, 0.16-0.69) and was consistent in short-term analyses. There were no significant differences between the 2 groups in the risks of gastrointestinal bleeding (HR, 0.99; 95% CI, 0.72-1.35), ischemic stroke (HR, 1.06; 95% CI, 0.86-1.31), or all-cause mortality (HR, 0.93; 95% CI, 0.80-1.09).

**Conclusion:**

In NVAF patients at nutritional risk, dabigatran was associated with a lower risk of ICH compared with rivaroxaban, without compromising effectiveness.

## Essentials

• Poor nutrition affects drug safety in NVAF.
• We studied 10,658 NVAF patients at nutritional risk using real-world medical data.
• Dabigatran showed a lower risk of intracranial hemorrhage than rivaroxaban.
• Dabigatran reduces intracranial hemorrhage risk while effectively preventing stroke.

## 1 Introduction

Atrial fibrillation (AF) is a major public health burden associated with an increased risk of ischemic stroke (IS) and systemic embolism [1,2]. Oral anticoagulation (OAC) remains the cornerstone of stroke prevention therapy [[1], [2], [3]]. However, the clinical benefit of OACs must always be weighed against the risk of bleeding, particularly intracranial hemorrhage (ICH), which has high morbidity and mortality rates [3,4]. This risk-benefit balance is particularly challenging to optimize in patients with significant comorbidities [5,6].

Malnutrition is a common but frequently overlooked comorbidity in patients with AF and has been identified as an independent predictor of poor prognosis [7]. Patients at nutritional risk are often characterized by hypoalbuminemia and frailty, conditions that are theoretically linked to both hypercoagulability and an increased bleeding risk [8]. Although non-vitamin K antagonist OACs (NOACs) have demonstrated noninferiority or superiority to warfarin in general AF populations [9,10], evidence regarding their comparative safety and effectiveness specifically in patients at nutritional risk remains limited. Current guidelines generally recommend NOACs over warfarin but do not prioritize specific agents for malnourished patients [1,2]. This lack of specific guidance is particularly relevant when considering dabigatran and rivaroxaban, 2 widely used NOACs that differ substantially in their pharmacologic characteristics. Rivaroxaban exhibits high plasma protein binding (>90%), whereas dabigatran has a low protein-binding fraction (∼35%) [11,12]. Based on these pharmacologic differences, we hypothesized that nutritional status may differentially influence the safety profiles of these 2 agents by impacting plasma protein levels.

To address this knowledge gap, we conducted a real-world cohort study to compare the safety and effectiveness of dabigatran with those of rivaroxaban in nonvalvular AF (NVAF) patients at nutritional risk. The primary aim was to evaluate whether the choice of anticoagulant is associated with differential risks of bleeding and ischemic events in this vulnerable population.

## 2 Methods

### 2.1 Study design and population

This new-user, active-comparator cohort study was conducted using real-world data from the Tianjin Health and Medical Data Platform (THMDP) and was reported in accordance with the REporting of studies Conducted using Observational Routinely-collected Data in Pharmacoepidemiology (RECORD-PE) guidelines. The THMDP is a comprehensive electronic health record database covering a population of approximately 14 million residents in Tianjin, China. It includes electronic health information from 97.8% (261/267) of community healthcare centers and 78 public hospitals, including 100% (42/42) of tertiary general hospitals and 100% (36/36) of secondary hospitals. The validity and utility of the THMDP for cardiovascular research have been previously established [[13], [14], [15], [16], [17], [18], [19], [20]]. The initial study population consisted of 134,516 participants who were newly prescribed an OAC during hospitalization between January 2015 and June 2020. To identify the target cohort, we applied a rigorous multistep screening process (Supplementary Figure 1). We excluded patients with OAC prescriptions in the preceding 12 months, those treated solely for venous thromboembolism prevention, and those with a diagnosis of trauma. To ensure diagnostic accuracy, we identified AF in our study based on a standard discharge diagnosis recorded during the index hospitalization event (or a prior hospitalization) in the THMDP. The discharge diagnosis was determined by reviewing specific International Classification of Diseases, 10th Revision (ICD-10) codes (I48), ensuring that patients included in our study had an AF diagnosis accurately documented as the primary indication for OAC initiation. Following AF confirmation, we further excluded patients with valvular heart disease or mechanical valve replacements, or those who initiated OACs more than 90 days after admission (this criterion was applied to ensure that baseline laboratory values remained temporally relevant and closely reflected the patient’s nutritional status at the time of OAC initiation). Given the study’s focus on nutritional status, patients with missing baseline albumin or lymphocyte data were excluded. Crucially, to define our study population as those at nutritional risk, we excluded patients with a Prognostic Nutritional Index (PNI) score > 50. Finally, after excluding users of warfarin or other OACs, the final cohort comprised 10,658 eligible patients (dabigatran *n* = 1819; rivaroxaban *n* = 8839). The study protocol was approved by the Ethics Committee of Tianjin Medical University General Hospital (IRB2022-YX-235-01), which waived the requirement for informed consent due to the observational, anonymized nature of the study.

### 2.2 Assessment of nutritional status and exposure

The nutritional status of each participant was evaluated at baseline using the PNI score, a validated indicator derived from serum albumin concentration and peripheral lymphocyte count [21]. The PNI score was calculated using the following formula: PNI = 10 × serum albumin value (g/dL) + 0.005 × total lymphocyte count (/μL). A PNI score ≤ 50 was used to define the presence of nutritional risk in this study. The severity of malnutrition was classified as mild (45 < PNI ≤ 50), moderate (40 < PNI ≤ 45), and severe (PNI ≤ 40) [[22], [23], [24]].

The primary exposure was initiation of specific NOACs. Patients were categorized into the dabigatran or rivaroxaban group based on their index prescription. Dosage regimens were stratified into 3 categories according to the prescribed daily dose: (1) standard dosage: dabigatran 150 mg twice daily or rivaroxaban 20 mg once daily; (2) low dosage: dabigatran 110 mg twice daily or rivaroxaban 15 mg (or 10 mg) once daily; (3) high dosage: any daily dose exceeding the standard regimens. The index time was defined as the date of the first prescription of either dabigatran or rivaroxaban.

### 2.3 Outcomes and follow-up

The study assessed clinical outcomes over a maximum follow-up period of 3 years. Bleeding outcomes included ICH (comprising intracerebral, subarachnoid, epidural, and subdural hemorrhage), gastrointestinal bleeding (GIB), and a composite bleeding outcome (defined as the occurrence of either ICH or GIB). Secondary outcomes included IS, acute myocardial infarction, and all-cause mortality. These outcomes were identified primarily using standard ICD-10 codes retrieved from the THMDP system. To ensure high diagnostic accuracy in this healthcare system, these standard ICD-10 discharge codes were strictly corroborated by objective clinical examinations, including computed tomography or magnetic resonance imaging for cerebrovascular events and endoscopy for GIB. Patient mortality data were primarily collected from the hospital electronic medical record system and death registration records from community healthcare centers. Follow-up began on the index date (ie, the date of the first OAC prescription) and continued until the first occurrence of a specific outcome, death, attainment of the maximum 3-year follow-up period (December 31, 2021), or discontinuation of the index OAC, whichever occurred first. To evaluate the effectiveness of the initial treatment strategy under real-world adherence patterns, our primary analysis incorporated an allowable grace period of 183 days; follow-up was right-censored exactly 183 days after the last prescription date if no new prescription of the index OAC was filled.

### 2.4 Study variables

The study’s baseline variables encompassed a broad range of demographic factors, clinical parameters, scoring scales, medical history, and medication use. Demographic factors included age, sex, current smoking status, and daily alcohol consumption, while laboratory assessments included serum albumin levels, lymphocyte counts, total cholesterol, and estimated glomerular filtration rate (eGFR). Scoring scales included the CHA_2_DS_2_-VASc (congestive heart failure, hypertension, age ≥ 75 years, diabetes mellitus, stroke/transient ischemic attack/thromboembolism, vascular disease, age 65-74 years, sex category), HAS-BLED (hypertension, abnormal renal/liver function, stroke, bleeding history or predisposition, labile international normalized ratio, elderly, drugs/alcohol concomitantly), and PNI scores. The medical history of patients was determined by searching for diagnoses recorded in the system prior to the inclusion date according to ICD-10 codes, including hypertension, diabetes mellitus, ischemic heart disease, stroke, heart failure, liver disease, and cancer. Furthermore, baseline medication use was defined as the presence of at least 1 relevant prescription record within 6 months prior to the index date, including antiplatelet agents, statins, angiotensin-converting enzyme inhibitors or angiotensin receptor blockers, β-blockers, calcium channel blockers, diuretics, and proton pump inhibitors.

### 2.5 Statistical analysis

Baseline characteristics were compared between the dabigatran and rivaroxaban groups. Normally distributed continuous variables were expressed as means ± SDs, whereas nonnormally distributed continuous variables were presented as medians with IQRs. Categorical variables were expressed as frequencies and percentages. To evaluate the balance of baseline characteristics between the dabigatran and rivaroxaban groups, we calculated standardized mean differences (SMDs) rather than using statistical hypothesis testing. An absolute SMD > 0.10 was considered indicative of a meaningful imbalance between the groups.

To validate the rationale for focusing on patients at nutritional risk, we first conducted an exploratory analysis of the entire cohort of eligible NVAF patients, including those with normal nutritional status (PNI > 50). Given that ICH is the most severe bleeding complication and is highly sensitive to systemic anticoagulation intensity, we employed a multivariable fractional polynomial interaction (MFPI) approach to investigate its relationship with the PNI score and anticoagulant type. Based on the analysis findings, which suggested a significant effect modification by nutritional status, we defined our primary analytical cohort as patients with a PNI score ≤50.

In this predefined cohort of patients at nutritional risk, a multivariable Cox proportional hazards regression model was used to estimate hazard ratios (HRs) and 95% CIs for the association between NOAC type and clinical outcomes. Specifically, for all nonfatal secondary outcomes, given the high mortality rate in this frail cohort, we used a cause-specific hazard approach (treating all-cause death as a right-censoring event) to estimate the causal treatment effect. For all-cause mortality, a standard multivariable Cox model was used. Potential confounders were selected *a priori* based on clinical relevance, existing literature, and a conceptual causal framework to address potential confounding by indication. The causal structure of the study was visualized using a directed acyclic graph (Supplementary Figure 2), which guided the selection of baseline confounders. Consequently, all multivariable Cox models were adjusted for the following baseline covariates: age, sex, eGFR, current smoking, daily alcohol consumption, and a medical history of stroke, ischemic heart disease, cancer, liver disease, hypertension, diabetes mellitus, and heart failure, as well as baseline use of statins, antiplatelet agents, angiotensin-converting enzyme inhibitors or angiotensin receptor blockers, β-blockers, calcium channel blockers, diuretics, and proton pump inhibitors. The proportional hazards assumption was verified using Schoenfeld residuals. To visualize temporal trends, cumulative incidence curves for nonfatal outcomes were generated using the Aalen–Johansen estimator to account for the competing risk of all-cause mortality, while the cumulative incidence of all-cause death was estimated using the Kaplan–Meier method. Incidence rates (IRs) per 1000 person-years were calculated for each outcome to quantify the clinical impact of the treatment effect. Furthermore, the absolute risk difference at 3 years for nonfatal outcomes was estimated using the Aalen–Johansen approach, while the standardized risk difference (SRD) was calculated through regression adjustment (G-computation) to account for baseline confounders.

To assess short-term safety profiles, particularly for ICH, analyses were performed at specific time intervals: 91 days, 183 days, and 1 year. Additionally, stratified analyses were conducted to compare bleeding risk between the 2 agents, specifically in the standard- and low-dosage groups. Subgroup analyses were conducted to explore potential effect modifiers of ICH. Stratification variables included age (<75 vs ≥75 years), sex, current smoking status, liver disease, hypertension, diabetes mellitus, heart failure, and serum albumin levels (<4 g/dL vs ≥4 g/dL). Interactions between treatment groups and subgroups were statistically tested.

Furthermore, to verify the robustness of the primary findings, a series of sensitivity analyses were performed: (1) a 1:1 nearest-neighbor propensity score matching (PSM) analysis targeting the average treatment effect on the treated, using a caliper width of 0.2 SDs of the logit of the propensity score to thoroughly balance baseline characteristics; (2) a secondary 1:1 PSM analysis (applying the same matching parameters) excluding patients with a controlling nutritional status score < 2 (another validated tool for assessing nutritional risk, derived by summing points assigned to specific values of serum albumin, total cholesterol, and total lymphocyte count [25], where a cumulative score of 0 to 1 indicates normal nutritional status); (3) a strict on-treatment analysis evaluating safety during active drug exposure, where follow-up was right-censored at the exact date of treatment crossover or any prescription lapse (without the 183-day grace period used in the primary analysis), thereby eliminating immortal time bias; (4) restricting the analysis to patients with moderate-to-severe malnutrition (PNI ≤ 45); (5) excluding patients with a non-Tianjin household registration to ensure data completeness; (6) excluding patients with a high bleeding risk (HAS-BLED score > 3) or eGFR < 60 mL/min/1.73 m^2^; and (7) systematically excluding patients with specific high-risk comorbidities, including ischemic heart disease, stroke, and cancer. All statistical analyses were performed using STATA version 18.0 (StataCorp LLC), and a 2-tailed *P* value < .05 was considered statistically significant.

## 3 Results

### 3.1 Patient characteristics

We identified 10,658 eligible NVAF patients at nutritional risk (Table 1). The dabigatran group exhibited a better nutritional profile, characterized by higher serum albumin levels, lymphocyte counts, and consequently higher PNI scores. Conversely, the rivaroxaban group had a higher proportion of patients with severe nutritional risk (PNI < 40). Regarding dosage, low-dose regimens were predominantly prescribed overall, but with a substantially higher utilization rate in the dabigatran group (SMD > 0.7). Clinically, the rivaroxaban group presented with a higher prevalence of diabetes, cancer, and liver disease, as well as a slightly higher HAS-BLED score and greater use of specific concomitant medications such as calcium channel blockers and proton pump inhibitors. Other demographic factors (eg, age and sex) and comorbidities were generally well balanced (SMD < 0.1) between the 2 groups. Regarding treatment persistence, 69.1% (1257/1819) of patients in the dabigatran group and 96.0% (8483/8839) in the rivaroxaban group remained adherent to their initial therapy throughout the follow-up period without switching or discontinuing.

**Table 1:** Baseline characteristics of nonvalvular atrial fibrillation patients at nutritional risk, stratified by anticoagulant type.

Characteristic	Total participants (*N* = 10,658)	Dabigatran (*n* = 1819)	Rivaroxaban (*n* = 8839)	SMD
**Patient demographics**				
Age (y), mean ± SD	72.0 ± 10.2	71.3 ± 9.6	72.0 ± 10.3	0.071
Male sex	5885 (55.2)	1022 (56.2)	4863 (55.0)	0.023
Current smoking	1353 (12.7)	238 (13.1)	1115 (12.6)	0.014
Daily alcohol consumption	688 (6.5)	96 (5.3)	592 (6.7)	0.060
**Laboratory test**				
Albumin (g/dL)	3.80 (3.50, 4.01)	3.84 (3.60, 4.03)	3.78 (3.49, 4.00)	0.203
Lymphocyte count (10^3^/uL)	1.30 (1.00, 1.65)	1.37 (1.07, 1.71)	1.29 (0.98, 1.64)	0.141
Total cholesterol (mg/dL)	157 (131.0, 186.0)	159 (133.0, 186.0)	157 (131.0, 186.0)	0.024
eGFR (mL/min/1.73 m^2^)	83.0 (67.2, 93.1)	83.9 (69.2, 93.2)	82.8 (66.6, 93.0)	0.066
**Clinical score**				
HAS-BLED score	1.0 (0.0, 2.0)	1.0 (0.0, 2.0)	1.0 (0.0, 2.0)	0.109
CHA_2_DS_2_-VASC score	4.0 (3.0, 5.0)	4.0 (3.0, 5.0)	4.0 (3.0, 5.0)	0.081
PNI score	45.0 (41.4, 47.7)	45.9 (42.7, 48.2)	44.8 (41.1, 47.5)	0.255
PNI score category				0.235
45-50	5326 (50.0)	1056 (58.1)	4270 (48.3)	
40-45	3328 (31.2)	537 (29.5)	2791 (31.6)	
<40	2004 (18.8)	226 (12.4)	1778 (20.1)	
**Previous history**				
Hypertension	8311 (78.0)	1445 (79.4)	6866 (77.7)	0.043
Diabetes mellitus	3091 (29.0)	400 (22.0)	2691 (30.4)	0.193
Ischemic heart disease	536 (5.0)	79 (4.3)	457 (5.2)	0.039
Stroke	1715 (16.1)	251 (13.8)	1464 (16.6)	0.077
Cancer	926 (8.7)	75 (4.1)	851 (9.6)	0.219
Heart failure	4662 (43.7)	837 (46.0)	3825 (43.3)	0.055
Liver disease	3006 (28.2)	441 (24.2)	2565 (29.0)	0.108
**Anticoagulant dosage category**				
Standard dosage	3614 (33.9)	161 (8.9)	3453 (39.1)	0.757
Low dosage	6931 (65.0)	1649 (90.7)	5282 (59.8)	0.766
High dosage	113 (1.1)	9 (0.5)	104 (1.2)	0.075
**Previous medication use**				
Statin	2865 (26.9)	455 (25.0)	2410 (27.3)	0.051
Antiplatelet therapy	3658 (34.3)	563 (31.0)	3095 (35.0)	0.087
ACEI/ARB	2330 (21.9)	350 (19.2)	1980 (22.4)	0.078
β-blockers	2762 (25.9)	463 (25.5)	2299 (26.0)	0.013
Calcium channel blockers	2421 (22.7)	346 (19.0)	2075 (23.5)	0.109
Proton pump inhibitors	1993 (18.7)	271 (14.9)	1722 (19.5)	0.122
Diuretic	1689 (15.8)	235 (12.9)	1454 (16.4)	0.100

Values are presented as mean ± SD, median (IQR), or *n* (%).

ACEI, angiotensin-converting enzyme inhibitor; ARB, angiotensin receptor blocker; CHA_2_DS_2_-VASc, congestive heart failure, hypertension, age ≥ 75 years, diabetes mellitus, stroke/transient ischemic attack/thromboembolism, vascular disease, age 65-74 years, sex category; eGFR, estimated glomerular filtration rate; HAS-BLED, hypertension, abnormal renal/liver function, stroke, bleeding history or predisposition, labile international normalized ratio, elderly, drugs/alcohol concomitantly; PNI, Prognostic Nutritional Index; SMD, standardized mean difference.

### 3.2 Effect of continuous nutritional status on anticoagulant-related ICH risk

**Figure 1:**
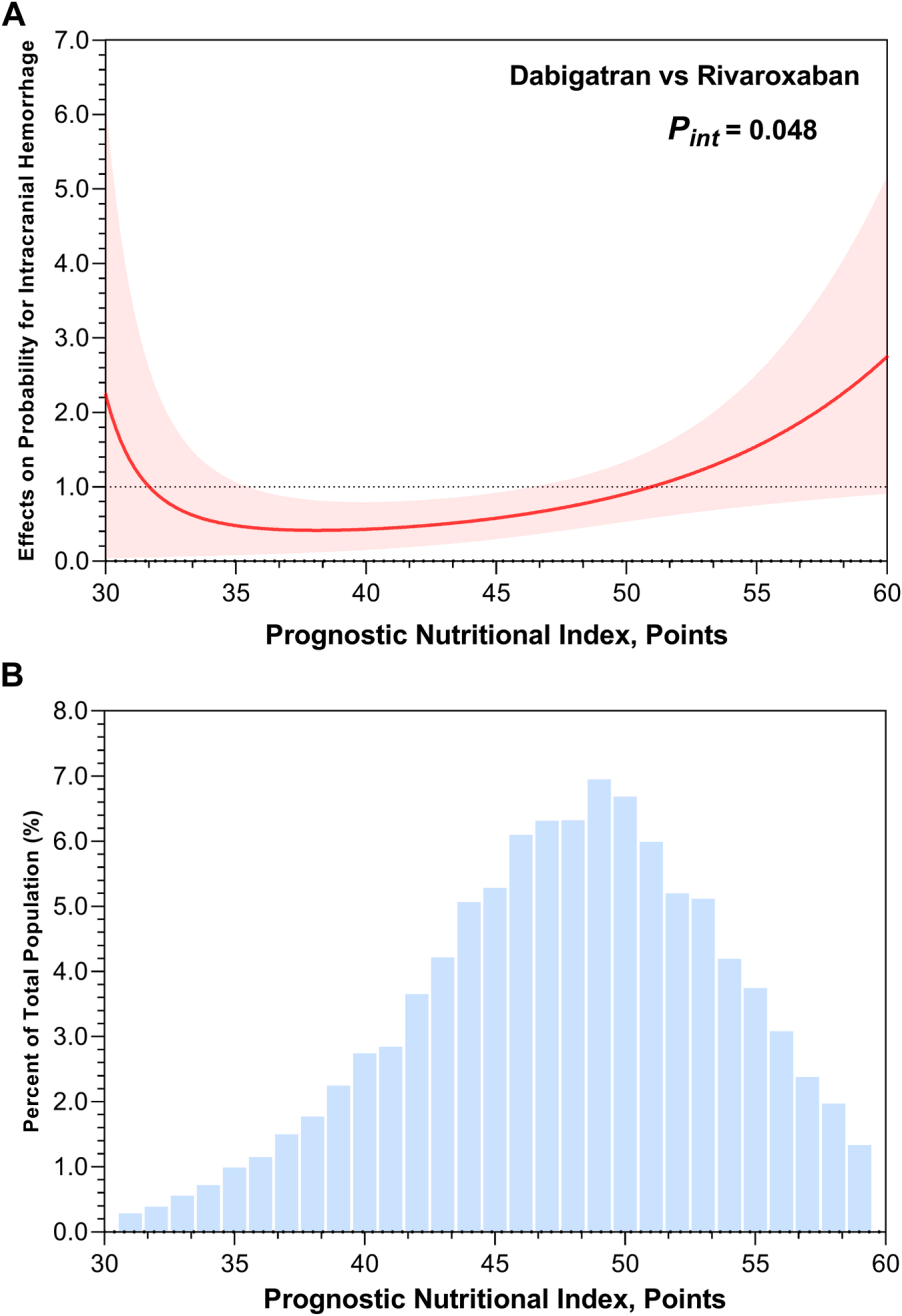
The effect of continuous nutritional status on anticoagulant-related intracranial hemorrhage risk. The figure shows the continuous relationship between the Prognostic Nutritional Index score and the comparative safety of dabigatran vs rivaroxaban for intracranial hemorrhage, derived from multivariable fractional polynomial interaction analysis. (A) The red solid line represents the estimated hazard ratio for dabigatran (with rivaroxaban as the reference), and the pink shaded area indicates the corresponding 95% CIs. (B) The histogram shows the percentage distribution of the study population across the Prognostic Nutritional Index score spectrum. The vertically aligned panels allow for direct comparison between the treatment effect and data density. *P*_int_, *P* value for interaction.

The MFPI analysis revealed a significant interaction between anticoagulant type and PNI score for ICH risk (*P* for interaction = .048). As illustrated in Figure 1, the safety benefit of dabigatran was most pronounced in patients with lower PNI scores. The protective effect attenuated as PNI scores increased, with the upper boundary of the 95% CI crossing the line of unity (HR, 1.0) as PNI scores approached 50. This data-driven finding supports the specific relevance of anticoagulant selection in the population defined by PNI ≤ 50.

### 3.3 Bleeding outcomes

**Figure 2:**
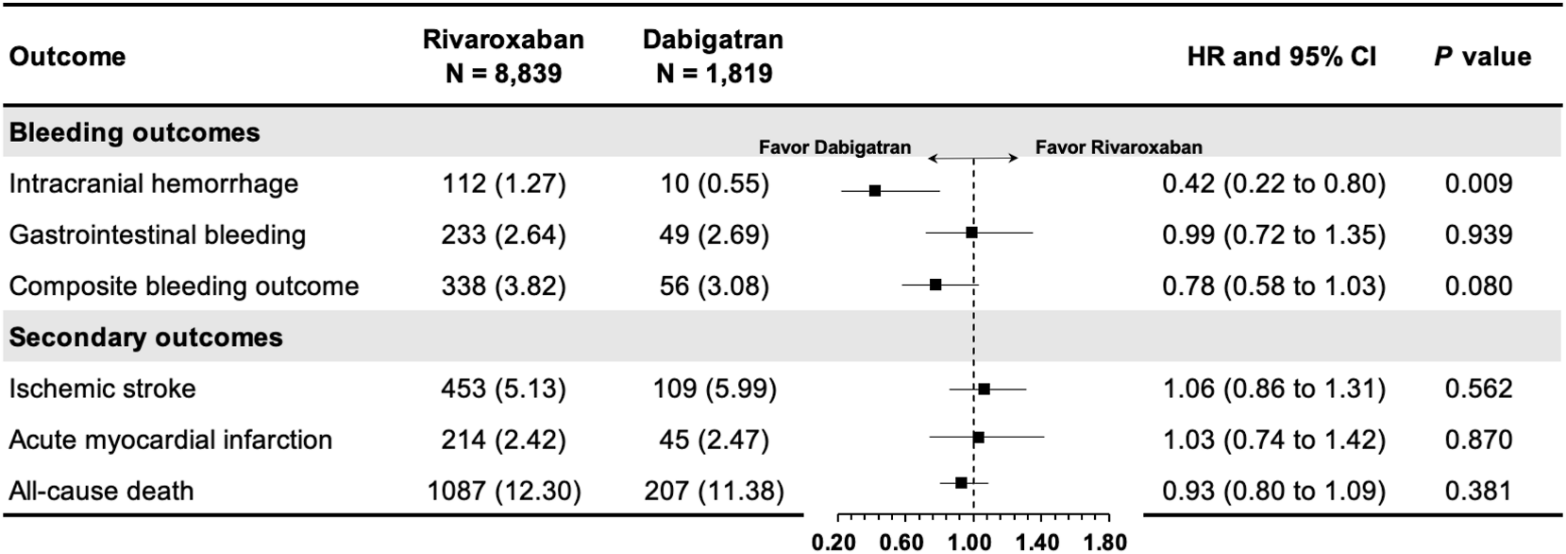
Comparative risks of bleeding and effective events between dabigatran and rivaroxaban. Forest plot showing the adjusted hazard ratios (HRs) and 95% CIs for bleeding and effective outcomes comparing dabigatran with rivaroxaban over a maximum follow-up of 3 years. The central squares represent the estimated HRs, and the horizontal lines indicate the 95% CIs. The model was adjusted for baseline characteristics, including age, sex, estimated glomerular filtration rate, current smoking, daily alcohol consumption, and a medical history of stroke, ischemic heart disease, cancer, liver disease, hypertension, diabetes mellitus, and heart failure, as well as baseline use of statins, antiplatelet agents, angiotensin-converting enzyme inhibitors or angiotensin receptor blockers, β-blockers, calcium channel blockers, diuretics, and proton pump inhibitors.

**Figure 3:**
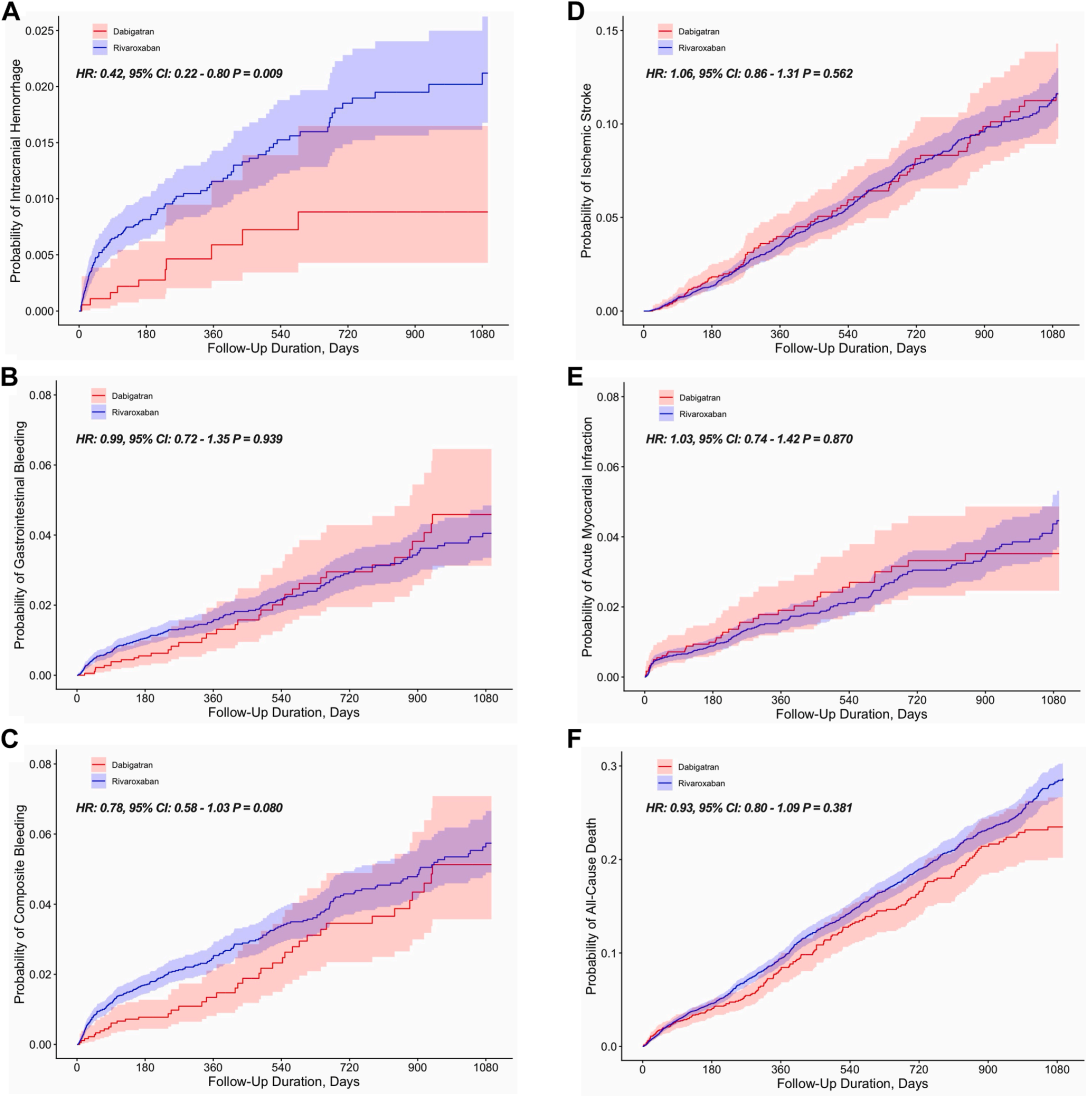
Adjusted cumulative incidence curves for clinical outcomes over 3 years. The images show the adjusted cumulative incidence estimates for (A) intracranial hemorrhage, (B) gastrointestinal bleeding, (C) the composite bleeding outcome, (D) ischemic stroke, (E) acute myocardial infarction, and (F) all-cause mortality. The red dashed line represents the dabigatran group, and the blue solid line the rivaroxaban group. The corresponding semitransparent shaded areas represent the 95% CIs for each group. The cumulative incidence for all nonfatal outcomes was estimated using the Aalen–Johansen approach to properly account for the competing risk of death. HR, hazard ratio.

Over a maximum follow-up period of 3 years, the incidence of ICH was 0.55% in the dabigatran group and 1.27% in the rivaroxaban group. The IR of ICH was 4.46 per 1000 person-years in the dabigatran group, lower than the 11.31 per 1000 person-years observed in the rivaroxaban group (Supplementary Table 1). After multivariable adjustment, dabigatran use was associated with a lower risk of ICH than rivaroxaban (HR, 0.42; 95% CI, 0.22-0.80; *P* = .009; Figures 2 and 3A). This safety benefit was further confirmed by absolute risk metrics, with a 3-year absolute risk difference of −1.24% (95% CI, −2.02% to −0.46%) and an SRD of −0.76% (95% CI, −1.16% to −0.36%; *P* < .001; Supplementary Table 2). This association was consistently observed in the short-term analyses at 91 days, 183 days, and 1 year (Supplementary Figures 3–6). Regarding other bleeding outcomes, no statistically significant differences were observed between the 2 groups (Figures 2 and 3B, C). There was no significant difference in the risk of GIB between dabigatran and rivaroxaban users (HR, 0.99; 95% CI, 0.72-1.35; *P* = .94). Similarly, the composite bleeding outcome did not differ significantly between the 2 groups (HR, 0.78; 95% CI, 0.58-1.03; *P* = .08).

### 3.4 Effectiveness and mortality

No statistically significant differences were observed between the 2 groups for effectiveness outcomes. The risk of IS was comparable between the dabigatran and rivaroxaban groups (HR, 1.06; 95% CI, 0.86-1.31; *P* = .56; Figure 2). Similarly, there was no significant difference in the risk of acute myocardial infarction (HR, 1.03; 95% CI, 0.74-1.42; *P* = .87). Furthermore, the risk of all-cause mortality did not differ significantly between the 2 groups (HR, 0.93; 95% CI, 0.80-1.09; *P* = .38). The cumulative incidence curves for these outcomes are shown in Figure 3D–F. IRs per 1000 person-years and absolute risk metrics for secondary outcomes are detailed in Supplementary Tables 1 and 2. No significant SRDs were observed between dabigatran and rivaroxaban for all secondary outcomes (Supplementary Table 2), indicating comparable effectiveness and survival after balancing baseline characteristics.

### 3.5 Dose-stratified analysis

In the dose-stratified analysis, low-dose dabigatran was associated with a lower risk of ICH than low-dose rivaroxaban (HR, 0.33; 95% CI, 0.16-0.69; *P* = .003). Among patients receiving the standard dosage, no statistically significant difference in ICH risk was observed between the 2 agents (HR, 0.97; 95% CI, 0.23-4.13; *P* = .97). These findings indicate that the safety difference in ICH in the overall cohort was largely attributable to outcomes in the low dosage category (Supplementary Figure 7).

### 3.6 Subgroup and sensitivity analyses

Subgroup analyses for ICH generally showed consistent results across most strata, including age, sex, current smoking, and comorbidities. Notably, in patients with albumin levels < 4 g/dL, dabigatran use was associated with a lower risk of ICH compared with rivaroxaban (HR, 0.34; 95% CI, 0.15-0.79). No significant interactions were observed between the treatment effect and these stratification variables (Table 2).

**Table 2:** Subgroup analyses of the risk of intracranial hemorrhage between dabigatran and rivaroxaban.

Subgroup	Rivaroxaban		Dabigatran		*P*_int_
	Event/*N* (%)	HR (95% CI)	Event/*N* (%)	HR (95% CI)	
Age (y)					
<75	57/5040 (1.13)	Reference	7/1109 (0.63)	0.54 (0.24-1.19)	.37
≥75	55/3799 (1.45)	Reference	3/710 (0.42)	0.28 (0.09-0.91)	
**Sex**					
Male	63/4863 (1.30)	Reference	5/1022 (0.49)	0.38 (0.15-0.94)	.74
Female	49/3976 (1.23)	Reference	5/797 (0.63)	0.45 (0.18-1.14)	
**Smoking**					
No	96/7724 (1.24)	Reference	7/1581 (0.44)	0.35 (0.16-0.75)	.24
Yes	16/1115 (1.43)	Reference	3/238 (1.26)	0.80 (0.23-2.82)	
**Liver disease**					
No	78/6274 (1.24)	Reference	8/1378 (0.58)	0.45 (0.22-0.93)	.69
Yes	34/2565 (1.33)	Reference	2/441 (0.45)	0.34 (0.08-1.42)	
**Hypertension**					
No	15/1973 (0.76)	Reference	1/374 (0.27)	0.28 (0.04-2.17)	.78
Yes	97/6866 (1.41)	Reference	9/1445 (0.62)	0.44 (0.22-0.87)	
**Diabetes**					
No	74/6148 (1.20)	Reference	7/1419 (0.49)	0.40 (0.18-0.87)	.73
Yes	38/2691 (1.41)	Reference	3/400 (0.75)	0.47 (0.14-1.51)	
**Heart failure**					
No	57/5014 (1.14)	Reference	5/982 (0.51)	0.44 (0.18-1.10)	.94
Yes	55/3825 (1.44)	Reference	5/837 (0.60)	0.40 (0.16-1.00)	
**Albumin < 4 g/dL**					
No	28/2450 (1.14)	Reference	4/603 (0.66)	0.56 (0.19-1.60)	.46
Yes	84/6389 (1.31)	Reference	6/1216 (0.49)	0.34 (0.15-0.79)	

The reference group refers to rivaroxaban. HRs were estimated using multivariable cause-specific Cox proportional hazards models. The models were adjusted for the following prespecified baseline confounders: age, sex, estimated glomerular filtration rate, current smoking, daily alcohol consumption, medical history (stroke, ischemic heart disease, cancer, liver disease, hypertension, diabetes mellitus, and heart failure), and baseline use of concomitant medications (statins, antiplatelet agents, angiotensin-converting enzyme inhibitors/angiotensin receptor blockers, β-blockers, calcium channel blockers, diuretics, and proton pump inhibitors).

HR, hazard ratio; *P*_int_, *P* value for interaction.

The primary findings remained robust across a series of sensitivity analyses (Supplementary Table 3). The favorable safety profile of dabigatran for ICH persisted in both the 1:1 PSM analysis and the strict on-treatment analysis. Consistent results were also observed when analyses excluded patients with a controlling nutritional status score < 2 (1:1 PSM), those with a non-Tianjin household registration, and those with a high bleeding risk (HAS-BLED score > 3). Furthermore, the results remained consistent after excluding patients with renal impairment as well as those with specific high-risk comorbidities. Notably, even in the subset of patients with moderate-to-severe malnutrition (PNI ≤ 45), the protective trend remained consistent with the main analysis.

## 4 Discussion

In this real-world cohort of NVAF patients at nutritional risk, we found that dabigatran was associated with a lower risk of ICH compared with rivaroxaban. This safety advantage was primarily observed in patients receiving low-dose regimens and was not accompanied by an associated increase in the risk of ischemic events or all-cause mortality.

Our findings are broadly consistent with and yet significantly extend the existing body of evidence in general AF populations [[26], [27], [28]]. For instance, a large-scale analysis of the U.S. Food and Drug Administration’s Mini-Sentinel database reported a lower risk of ICH with dabigatran than with rivaroxaban among Medicare beneficiaries [29]. These studies provide crucial evidence, indicating a potential drug-specific difference in cerebral bleeding safety. However, few studies have specifically targeted nutritionally compromised patients, in whom altered pharmacokinetics and frailty may lead to divergent safety outcomes compared with the general population [30]. Our study addresses this critical gap, indicating that the favorable safety profile of dabigatran for ICH extends to this specific high-risk population. By confirming this safety advantage in a cohort defined by nutritional risk, our results provide evidence that the pharmacologic properties of dabigatran may be particularly favorable in patients with compromised physiological reserve.

The lower risk of ICH associated with dabigatran use in this cohort is likely explained by its distinct pharmacologic profile. Rivaroxaban is highly protein-bound to albumin [12]. In malnourished patients with hypoalbuminemia, the unbound active fraction of rivaroxaban may unpredictably increase, potentially elevating the bleeding risk in vulnerable cerebral vessels [31]. Conversely, dabigatran’s low protein binding makes its anticoagulant effect largely independent of albumin levels, suggesting greater pharmacokinetic stability in patients with nutritional risk. This hypothesis is supported by our MFPI analysis of the total population. We observed a significant interaction in which the safety advantage of dabigatran was evident in patients with lower PNI scores but diminished as nutritional status normalized (PNI > 50). This “dose-response” relationship between nutritional status and drug safety provides robust, data-driven validation of our study’s focus on the PNI ≤ 50 subgroup. The subgroup analysis supports this pharmacokinetic mechanism, revealing a particularly protective effect of dabigatran in patients with albumin levels < 4 g/dL. The observed dosage patterns further reinforce this finding. The predominant use of low-dose dabigatran (90.7% of users), a regimen with a well-established safety advantage in the randomized evaluation of long-term anticoagulant therapy trial [32], likely amplified the safety benefits in this high-risk, nutritionally compromised population.

An interesting finding of our study is the observed risk difference for ICH but not for GIB. This discrepancy likely reflects the distinct pathophysiological mechanisms and pharmacologic influences driving these 2 bleeding events. The risk of ICH is primarily influenced by systemic anticoagulation intensity and the integrity of the cerebral microvasculature [33]. In this context, the lower risk associated with dabigatran likely reflects a more favorable systemic safety profile, possibly due to its stable pharmacokinetics in patients with hypoalbuminemia. In contrast, the risk of GIB is a composite of both systemic anticoagulation and local drug effects within the gastrointestinal tract [34,35]. Dabigatran etexilate, the prodrug, undergoes hydrolysis to its active form in the gut. Unabsorbed drug and its metabolites can remain in the intestinal lumen, potentially exerting a direct topical anticoagulant effect on the mucosa or causing nonspecific irritation [32]. This local gastrointestinal effect could counteract the benefit of a lower systemic bleeding risk, resulting in a GIB incidence comparable to that of rivaroxaban. Therefore, our results suggest that the safety advantage of dabigatran in malnourished patients is specific to ICH, a site highly sensitive to systemic effects, whereas GIB risk is modulated by a combination of both local and systemic actions [31].

Our findings offer direct guidance on anticoagulant selection in the growing NVAF population at nutritional risk. For these vulnerable individuals, dabigatran, particularly at a low dosage, may be a preferable therapeutic option to rivaroxaban to minimize the risk of ICH without compromising stroke prevention [36]. Furthermore, this study highlights the necessity of integrating nutritional assessment, such as the PNI or controlling nutritional status score [25], into routine risk stratification for NVAF patients. Such an approach can help identify those who would benefit most from a tailored anticoagulation strategy, thereby moving beyond a one-size-fits-all approach and enhancing personalized care in clinical practice [37].

This study has several strengths. First, to our knowledge, this is the first and largest study to provide a head-to-head comparison of dabigatran and rivaroxaban, specifically in the high-risk subgroup of NVAF patients at nutritional risk (PNI ≤ 50). Second, the use of a large, real-world cohort enhances the generalizability of our results to routine clinical practice. Third, the robustness of our primary findings was rigorously tested through a comprehensive series of subgroup and sensitivity analyses, including PSM, which supports the validity of the observed associations. Nevertheless, several limitations should be acknowledged. First, the observational nature of this cohort study inherently limits our ability to establish causality and is susceptible to unmeasured confounding. Factors such as frailty indices, medication adherence, and dynamic changes in nutritional status over time were not available for analysis. Second, to avoid misclassification bias related to our cohort-defining inclusion criterion (the PNI score), patients with missing baseline albumin or lymphocyte data were excluded. This complete-case approach, however, may introduce selection bias and slightly limit the generalizability of our findings. Third, the reliance on ICD-10 codes for identifying comorbidities and outcomes may be subject to misclassification bias. Lastly, while we stratified data by dosage categories, we could not account for dose adjustments during the follow-up period, which may have influenced the outcomes. Therefore, future prospective studies are warranted to confirm these findings. Ultimately, randomized controlled trials specifically designed for the NVAF population are needed to establish an optimal anticoagulation strategy for malnourished patients.

## 5 Conclusion

In NVAF patients at nutritional risk, dabigatran was associated with a reduced risk of ICH compared with rivaroxaban, particularly in those receiving low-dose regimens. Furthermore, dabigatran maintained comparable effectiveness in stroke prevention and survival. These findings support dabigatran as a preferred anticoagulant strategy for optimizing the safety-efficacy balance in this vulnerable population.
