# Supplementary material for: Safety and effectiveness of dabigatran vs rivaroxaban in nonvalvular atrial fibrillation patients at nutritional risk
Source: Res Pract Thromb Haemost. 2026 Jul 21;10(5):106865. doi: 10.1016/j.rpth.2026.106865 (PMC13485655; doi:10.1016/j.rpth.2026.106865)
Supplement: Supplementary file 1 [file mmc1.docx]

**Supplementary material**

Figure S1. Flow Chart of Study.

Figure S2. Directed Acyclic Graph of the Causal Framework.

Figure S3. Short-term Risk of Intracranial Hemorrhage at Specific Time Intervals.

Figure S4. Cumulative Incidence of Intracranial Hemorrhage at 91 Days.

Figure S5. Cumulative Incidence of Intracranial Hemorrhage at 183 Days.

Figure S6. Cumulative Incidence of Intracranial Hemorrhage at 1 Year.

Figure S7. Risk of Intracranial Hemorrhage Stratified by Dosage Regimen.

Table S1. Incidence Rates of Clinical Outcomes per 1000 Person-Years.

Table S2. Absolute and Standardized Risk Differences of Clinical Outcomes at 3 Years.

Table S3. Sensitivity Analyses for the Association Between Anticoagulant Type and Intracranial Hemorrhage.

**Figure S1**. Flow Chart of Study

**Figure S2**. Directed Acyclic Graph of the Causal Framework

**Figure S3**. Short-term Risk of Intracranial Hemorrhage at Specific Time Intervals.

Forest plot displaying the hazard ratios (HRs) and 95% confidence intervals (CIs) for intracranial hemorrhage comparing Dabigatran versus Rivaroxaban at 1 year, 183 days, and 91 days. **Abbreviations**: CI, confidence interval; HR, hazard ratio.

**Figure S4**. Cumulative Incidence of Intracranial Hemorrhage at 91 Days.

**Figure S5**. Cumulative Incidence of Intracranial Hemorrhage at 183 Days.

**Figure S6**. Cumulative Incidence of Intracranial Hemorrhage at 1 Year.

**Figure S7**. Risk of Intracranial Hemorrhage Stratified by Dosage Regimen.

**Table S1. Incidence Rates of Clinical Outcomes per 1000 Person-Years**

| **Outcome** | **Dabigatran** | **Rivaroxaban** |
| --- | --- | --- |
| **Bleeding outcomes** | | |
| Intracranial hemorrhage | 4.46 (2.40 to 8.29) | 11.31 (9.40 to 13.61) |
| Gastrointestinal bleeding | 22.10 (16.70 to 29.24) | 23.67 (20.81 to 26.91) |
| Composite bleeding outcome | 25.73 (19.80 to 33.43) | 35.10 (31.55 to 39.05) |
| **Secondary outcomes** | | |
| Ischemic stroke | 50.57 (41.92 to 61.01) | 47.39 (43.22 to 51.96) |
| Acute myocardial infarction | 19.65 (14.67 to 26.32) | 21.02 (18.38 to 24.03) |
| All-cause death | 88.78 (77.47 to 101.74) | 104.90 (98.84 to 111.32) |

Incidence rates were calculated as the number of events per 1000 person-years of follow-up.

**Table S2.** **Absolute and Standardized Risk Differences of Clinical Outcomes at 3 Years**

| **Outcome** | **Absolute Risk Difference, % (95% CI)** | **Standardized Risk Difference, % (95% CI)** | ***P* Value (for SRD)** |
| --- | --- | --- | --- |
| **Bleeding outcomes** | | | |
| Intracranial hemorrhage | -1.24 (-2.02 to -0.46) | -0.76 (-1.16 to -0.36) | <0.001 |
| Gastrointestinal bleeding | 0.54 (-1.29 to 2.36) | 0.04 (-0.80 to 0.87) | 0.931 |
| Composite bleeding outcome | -0.61 (-2.57 to 1.35) | -0.80 (-1.70 to 0.10) | 0.082 |
| **Secondary outcomes** | | | |
| Ischemic stroke | -0.02 (-2.89 to 2.84) | 1.03 (-0.22 to 2.29) | 0.107 |
| Acute myocardial infarction | -0.94 (-2.39 to 0.51) | 0.16 (-0.66 to 0.98) | 0.706 |
| All-cause death | -5.18 (-8.88 to -1.48) | 0.58 (-1.18 to 2.35) | 0.519 |

Risk differences were calculated as the risk in the dabigatran group minus the risk in the rivaroxaban group. A negative value indicates a lower absolute risk associated with dabigatran. Absolute Risk Difference: Represents the crude difference in 3-year cumulative incidence estimates. For all non-fatal outcomes, these estimates were derived using the Aalen-Johansen method to properly account for the competing risk of mortality, while the Kaplan-Meier method was used for all-cause mortality. Standardized Risk Difference: Estimated using regression adjustment (G-computation) to account for baseline confounders. Abbreviations: ARD, absolute risk difference; CI, confidence interval; SRD, standardized risk difference.

**Table S3.** Sensitivity Analyses for the Association Between Anticoagulant Type and Intracranial Hemorrhage.

|  | **Rivaroxaban** | **Dabigatran** | **HR, 95% CI** | ***P* value** |
| --- | --- | --- | --- | --- |
| **1:1 Propensity Score Match** | |  |  | |
|  | 22/1819 (1.21) | 10/1819 (0.55) | 0.44 (0.21 to 0.92) | 0.030 |
| **Excluding Patients With** **CONUT Score < 2 (1:1 PSM)** | | | | |
|  | 23/1497 (1.54) | 11/1497 (0.73) | 0.45 (0.22 to 0.93) | 0.030 |
| **Strict On-treatment Analysis (censoring at exact date of crossover or prescription lapse)** | | | | |
|  | 111/8839 (1.26) | 9/1819 (0.49) | 0.40 (0.20 to 0.79) | 0.008 |
| **Excluding Patients With** **Non-Tianjin Household Registration** | | | | |
|  | 106/7974 (1.33) | 9/1572 (0.57) | 0.41 (0.21 to 0.81) | 0.010 |
| **Limited to Patients With** **Moderate to Severe Malnutrition (PNI ≤ 45)** | | | | |
|  | 67/4569 (1.47) | 5/763 (0.66) | 0.41 (0.16 to 1.01) | 0.053 |
| **Excluding Patients With HAS-BLED Score > 3** | | | | |
|  | 107/8671 (1.23) | 10/1793 (0.56) | 0.44 (0.23 to 0.84) | 0.012 |
| **Excluding Patients With eGFR < 60 mL/min/1.73 m²** | | | | |
|  | 86/7224 (1.19) | 6/1547 (0.39) | 0.31 (0.14 to 0.72) | 0.006 |
| **Excluding Patients With Ischemic Heart Disease** | | | | |
|  | 104/8382 (1.24) | 10/1740 (0.57) | 0.45 (0.23 to 0.85) | 0.015 |
| **Excluding Patients With Stroke** | | | | |
|  | 87/7375 (1.18) | 9/1568 (0.57) | 0.47 (0.24 to 0.94) | 0.033 |
| **Excluding Patients With Cancer** | | | | |
|  | 104/7988 (1.30) | 10/1744 (0.57) | 0.44 (0.23 to 0.84) | 0.013 |
